# A Highly Reliable Embedded Optical Torque Sensor Based on Flexure Spring

**DOI:** 10.1155/2018/4362749

**Published:** 2018-04-15

**Authors:** Yuwang Liu, Tian Tian, Jibiao Chen, Fuhua Wang, Defu Zhang

**Affiliations:** ^1^State Key Laboratory of Robotics, Shenyang Institute of Automation, Chinese Academy of Sciences, Shenyang, China; ^2^Department of Mechanical Engineering, Shenyang Ligong University, Shenyang, China; ^3^Department of Mechanical Engineering, Northeastern University, Shenyang, China

## Abstract

We propose a new highly reliable and lightweight embedded optical torque sensor for biomimetic robot arm enabling the torque measurement in joints, which can measure torque of the joint by detecting torsion of its elastic element (mechanical structure or flexure element). Flexure spring is introduced as the elastic element of the torque sensor in this paper. Because of its curve modeling, flexure spring is not inclined to be broken contrast to crossbeam structure, which is commonly used in torque sensor. Thanks to this structure, we can build a torque sensor as an extremely compact and highly reliable size. Six types of flexure spring are proposed to be used as the elastic element of the torque sensor in this paper, which have the potential for the requirements of measurement range and multidimensional detection. The optical electronic, less influenced by electromagnetic interferences, is selected to measure the torsion displacement of the flexure spring. The proposed design is analyzed, which can obtain the successful measurement of the torque with a load capacity of 1 Nm. One of the designed optical torque sensors is optimized by FEM. The calibration and experiment are conducted to ensure its feasibility and performance.

## 1. Introduction

This paper focuses on the design of a new highly reliable and lightweight embedded optical torque sensor for biomimetic robots in order to ensure accuracy and stability while performing practical tasks of physical interaction with unstructured environment. Biomimetic robot arm is used to realize dexterous tasks such as interaction with human or manipulation with objects in hazardous conditions. To achieve the stability in contact with environment and realize safety while performing tasks of physical interaction with human and unstructured environment, it is essential to calculate the torque of each joint of the robot arm. The torque information of the torque sensor greatly enhances the force perception ability of biomimetic robot joint. The development of embedded torque sensor, especially, a lightweight and highly reliable one, has been of great interest for decades [1–3]. Based on its measurement methods, the torque sensor can be classified as strain gauges [4–6], capacitive [7–9], piezoelectric [10, 11], photoelectric [1, 12–17], and so on [18, 19].

Kang et al. [4] designed a mechanically decoupled six-axis force/torque sensor. It is a crossbeam sensor, which use strain gauge technique to detect force/moment. The size of this sensor is small with thickness of 37 mm, and the maximum torque detected by this sensor is 40 Nm. Also, Ma et al. [5] presented three kinds of compact torque sensors using strain gauge technique. The structure of these sensors is cartwheel flexure, which is also crossbeam. The measurement ranges of these sensors are 7 Nm, 22 Nm, and 50 Nm, respectively. The thickness is about 6 mm which is much suitable for using an embedded application. However, it is difficult to attach the strain gauge to the structure reliably while developing the type of torque sensor which is commonly sensitive to electrical noise and temperature. Kim et al. [7] designed a six-axis force/moment capacitive sensor using dielectric elastomer. Its deformation results in variation of capacitance, which is used for sensing force/moment. The size of this sensor is 67 mm × 30 mm, and the measurement range is 0.16 Nm. However, it is generally nonlinearity. Liu et al. [10] designed a no-elastic six-axis force/moment sensor using piezoelectric. The maximum torque detected by this sensor is as high as 250 Nm when the size is small as 50 mm × 30 mm. However, piezoelectric sensor is generally too stiff for this condition.

Recently, lots of researchers have been interested in this type of optical for its compact size, noncontact approach, and less electromagnetic interference. Kim et al. [12] designed a three-axis force/moment optical sensor using crossbeam which has a thickness of 7 mm and a diameter of 28 mm making it the most compact. Also, Shams et al. [13] presented an optical crossbeam torque sensor. This sensor is compact with a thickness of 11 mm and a diameter of 80 mm. Tsetserukou et al. [14] proposed three types of optical torque sensors. The first one is the cross-shaped spring with a diameter and thickness of 30 mm which can be used to detect 1.75 Nm load. The second one is the hub spoke-shaped spring with a diameter of 42 mm and thickness of 6.5 mm, and the sensor can be used to measure torque up to 0.8 Nm. The third one is the ring-shaped spring with a diameter of 42 mm and thickness of 10 mm, which can be used to handle torque up to 0.8 Nm.

Relatively speaking, the optical torque sensors tend to acquire much more deformation which is very suitable to build a biomimetic robot. However, the proposed sensors are commonly close to the yield stress at the maximum of torque size in which the overload protection needs to be added to keep it operating. However, it is inconvenient to build a sensor with this mechanism as it will increase the joint size. Factor of safety is a property to evaluate the ability of resistance to overload. It suggests the property of reliability, which is equal to the yield stress divided by the allowed one. However, an ideal compact size of the structure with a high factor of safety especially for the embedded sensor is very hard to acquire. The fact is that the factor of safety of optical torque sensors is generally limited to 1.0~2.0 [1, 14, 15, 34].

Flexure spring, also called planer spring or torsion spring, has been used in many fields for its advantages of compact structure and highly reliable performance [20–25]. It has been used to design a strain gauge sensor [22, 23]. However, the application of flexure spring to torque sensor has not been developed yet. In addition, limited to the mount space, there is also a challenge to build an optical torque sensor based on the spring.

**Figure 1:**
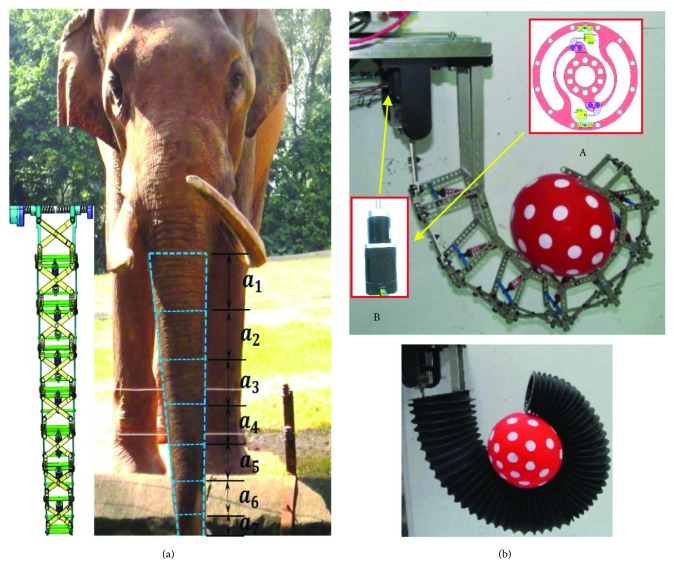
Elephant's trunk robot and torque sensor. (a) Elephant's trunk robot; (b) application of torque sensor in biomimetic actuation.

In this paper, we develop a highly reliable embedded optical torque sensor using flexure spring. The size of the torque sensor is designed to be extremely small, according to that of joint with the output of 1 Nm. The flexure spring, with the ability of storing energy and reducing the potential of stress concentration, is applied to realize the high reliability of torque sensor. In [33], we imitate an elephant's trunk with the special function of holding objects and propose a new type of shape-adaptive elephant's trunk robot to which the embedded optical torque sensor could be well applied. As shown in Figure 1, the elephant's trunk robot could be seen as a biomimetic elephant's trunk, whose effect could be equivalent to a kind of large artificial muscle. The robot consists of a base and some under-actuated joint units; the size of which is very similar to the real trunk. In an unstructured environment, the robot can adaptively grab various irregular-shaped objects and has flexible obstacle avoidance performance. *a*_*i*_ (*i* = 1~7) are under-actuated joint units in the robSSot. Each under-actuated joint unit is made up of a scissor mechanism, an elastic device, bottom links, and support links. The robot is directly driven by a single motor. Therefore, the output torque of the driving motor has a great influence on the holding stability of the robot. In Figure 1, (a) is the torque sensor and (b) is the driving motor. The inner ring of the sensor is fixed with the driving motor, and the outer ring is fixedly connected with the robot. The torque sensor we designed can accurately measure the output torque of the motor. Therefore, the torque sensor can be well applied to a biomimetic actuation similar to the elephant's trunk robot and is of great significance for studying the stability and performance characteristics of a bionic robot system.

Six types of flexure spring are proposed to be used as the mechanical structure. And the proper optical electronic is selected to measure the torsion of the flexure spring. As a result, six typical torque sensors are designed in Section 2. Simulations of all the six types of torque sensor were done by FEM (finite element method) software ANSYS, which are described in Section 3. Simulation results show that 2-rib torque sensor under 1 Nm load covers the measurement range of the selected optical electronic perfectly. The 2-rib torque sensor is selected to be further studied. And topology optimization of this sensor is represented in Section 3. The 2-rib optical torque sensor is calibrated, and several experiments (linearity, hysteresis, and repeatability) are conducted in Section 4.

## 2. Sensor Design

**Figure 2:**
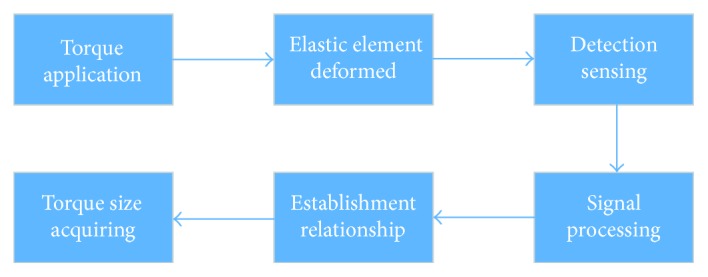
Working principle of torque sensor.

Torque sensor can be divided into two main parts, elastic element and sensing element. Elastic element produces torsion under the load of torque. The deformation accuracy is detected and measured by the sensing element (Figure 2). Thus, the applied torque could be acquired by establishing the relationship between the torque and the torsion.

### 2.1. Sensing Element Design

As the input of torque sensor is usually connected to motor [26, 27], the optical technique less influenced by electrical noise is preferred. In many types of optical detection, we decided to use ultrasmall size of photointerrupter as sensitive element to measure the relative motion of sensor's component. In those products, RPI-131 and RPI-121 are considered as their wide measurement range and small size. Although its size is slightly large, we finally selected RPI-131 to obtain a wide measurement range. The dimension of selected photointerrupter is 4.2 mm × 4.2 mm × 5.2 mm, the measurement range is about 0.6 mm, and its weight is only 0.05 g.

### 2.2. Elastic Element Design

The performance of elastic element widely varies according to its material and configuration.

#### 2.2.1. Material

Steel, aluminum, and titanium are general materials used in the design of torque sensor. Although the yield stress of aluminum is the smallest, we select it because its density is half of steel and 2/3 of titanium. Also, aluminum is the most economical among these materials. As a result, the aluminum 7075 with yield of 500 MPa is selected to manufacture the elastic element of torque sensor.

#### 2.2.2. Configuration

Two types of shapes, namely, axis structure and crossbeam, are generally used in elastic element design. The axis structure is usually used to load big torque because of its highly reliable performance and its capability to reduce coupling [3, 14, 28, 29]. However, it is difficult to be assembled into small space of a joint. On the other hand, the crossbeam is widely used because of its compact structure and good linearity [1, 4, 5, 7, 12–16]. However, it tends to produce stress concentration and even fail.

Flexure spring has been gradually applied in many fields such as cryocoolers [20, 21], sensors [22–24], and geophones [25] because of its compact structure and highly reliable performance. Contrast to the crossbeam, this structure could be more flexible which is beneficial to the requirement of the relatively wide deformation. In this section, the elastic element design is configured based on the flexure spring.

Because of the variety of the number and angle of ribs, the configuration would have potential for different measurement ranges. Theoretically, the number of ribs could be any. However, one to six ribs are considered feasible. The designed parameters of the torque sensor are mainly determined by the robot joint, whose maximum output torque is 1 Nm. These parameters are shown in Table 1. And the six typical configurations are shown in Table 2.

### 2.3. Integration

The interrupter shield and bracket were designed to vary the intensity of light and support the photointerrupter, respectively. The interrupter shield is attached to rib while bracket is attached to outer ring (shown in Table 2). Obviously, we can find that the multirib configurations have a wide measurement range and multidetection, while the sensitivity is much lower.

## 3. Analysis

In this section, the stiffness equations of the proposed configurations are deduced. With FEM simulation using the software of ANSYS, the theory is examined by building the relationship between design angle and stiffness. The optimal structures in these six typical configurations are selected according to their analysis results. Through the optimization of topology also using ANSYS, we finally acquired the ideal configuration.

### 3.1. Physical Modeling

The stiffness *k* means the required torque to produce a unit angle and can be calculated from

(1) $$\begin{array}{c} k=T/\Delta \theta ={TR}_{\text{out}}/\Delta s, \end{array}$$

where *T* is the applied torque, Δ*θ* is the deformed revolving angle, *R*_out_ is the radius of out flange, and Δ*s* is the produced tangential displacement. While *T* and *R*_out_ are given, the core issue is to acquire the formula of torsion in these sensors. To complete it, several assumptions and simplified physical model are needed. Assumptions

1. during the deformation, the cross section remains initial conditions;
2. for the selected Al-7075, the material of the beam is homogeneous;
3. when the deformation is small, the calculation can be seen as linearly elastic.

**Figure 3:**
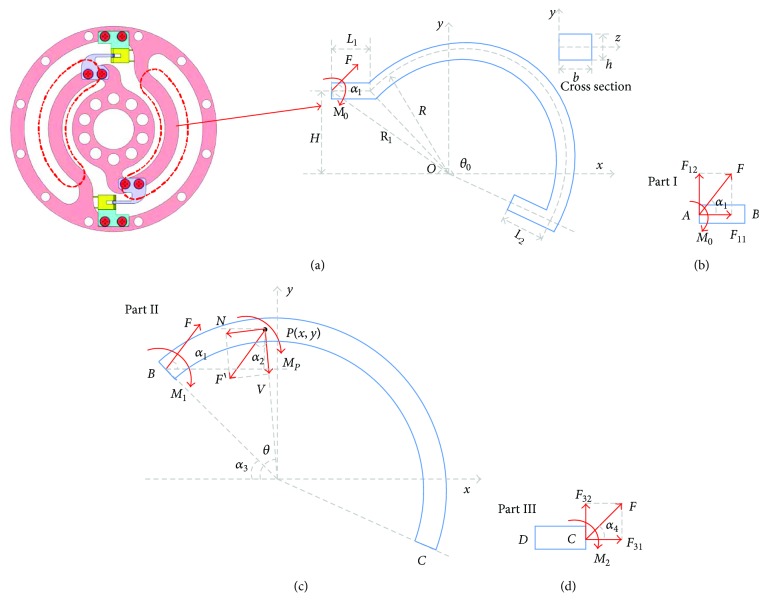
Physical model of the torque sensor: (a) overall appearance; (b) part I; (c) part II; (d) part III.

The built physical model can be seen in Figure 3.

The work done in deflecting the sensor Δ*θ* by torque *T* is 1/2*T*Δ*θ*, and for these parts, the stored energies due to torsion are *U*_1_, *U*_2_, and *U*_3_. Therefore,

(2) $$\begin{array}{c} U=U_{1}+U_{2}+U_{3}=\frac{1}{2}T\Delta \theta . \end{array}$$

In this model, we accept the equivalent force *F* and moment *M*_0_ stand for the applied torque *T*. They are

(3) $$\begin{array}{c} F=\frac{T}{{NR}_{\text{out}}},\\ M_{0}=F\left(R_{\text{out}}-R_{1}\right), \end{array}$$

where *N* denotes the number of ribs. For part I, we can calculate the normal force *F*_11_ and tangential force *F*_12_ as

(4) $$\begin{array}{c} F_{11}=F\cos\alpha_{1},\\ F_{12}=F\sin\alpha_{1}, \end{array}$$

where *α*_1_ is the angle between *F* and horizontal. Therefore, the stresses are

(5) $$\begin{array}{c} \sigma_{1x}=\frac{F_{11}}{A}+\frac{F_{12}xy}{I}+\frac{M_{0}y}{I},\\ \sigma_{1y}=0,\\ \tau_{1xy}=\frac{3}{2}\frac{F_{12}}{A}\left\{1-{\left(\frac{2y}{b}\right)}^{2}\right\}, \end{array}$$

where *A* is the sectional area, which can be calculated as *A* = *b* · *h* (*h* is the thickness and *b* is the wide). *I* is the moment of inertia, which is equal to *b*^3^*h*/12. Due to

(6) $$\begin{array}{c} U=\int \frac{1}{2}\sigma \varepsilon dV, \end{array}$$

where *ε* is the strain displacement. We can acquire the energy of part I as

(7) $$\begin{array}{c} U_{1}=\int \frac{1}{2}\sigma_{1}\varepsilon_{1}dV=\int \frac{\sigma_{1x}^{2}}{2E}dV+\int \frac{\tau_{1xy}^{2}}{2G}dV=\int {\left(\frac{F_{11}}{A}+\frac{F_{12}xy}{I}+\frac{M_{0}y}{I}\right)}^{2}\frac{1}{2E}dV+\int \frac{9}{8}\frac{F_{12}^{2}}{{GA}^{2}}{\left\{1-{\left(\frac{2y}{b}\right)}^{2}\right\}}^{2}dV=\frac{F_{11}^{2}L_{1}}{2EA}+\frac{F_{12}^{2}L_{1}^{3}}{6EI}+\frac{M_{0}^{2}L_{1}}{2EI}+\frac{F_{12}M_{0}L_{1}^{2}}{2EI}+\frac{3F_{12}^{2}L_{1}}{5GA}, \end{array}$$

where *E* is the modulus of elasticity, *G* is the modulus of rigidity, *L*_1_ is the distance between point *A* and *B*(*x*_1_, *y*_1_). For part II, in any point *P*(*x*_2_, *y*_2_) of the ribs, it has

(8) $$\begin{array}{c} V=F\cos\alpha_{2},\\ N=F\sin\alpha_{2},\\ M_{P}=-F_{11}\left(y_{2}-y_{1}\right)+F_{12}\left(x_{2}-x_{1}\right)+M_{1}, \end{array}$$

where *V*, *N*, and *M*_*P*_ are the tangential force, normal force, and moment size in point *P*, respectively. *α*_2_ is the angle between *F* and *V*, which can be calculated as *α*_2_ = *pi* − *θ* − *α*_1_. *M*_1_ is the moment in point *B*, which is equal to *M*_0_ + *F*_12_*L*_1_. As the rib is the curve beam, the expression according to the method of energy is given in [30]

(9) $$\begin{array}{c} U_{2}=\int \frac{M_{P}^{2}}{2AEe}d\theta +\int \frac{{kV}^{2}R}{2AG}d\theta +\int \frac{N^{2}R}{2AE}d\theta -\int \frac{M_{P}N}{AE}d\theta \end{array}$$

or

(10) $$\begin{array}{c} U_{2}=U_{21}+U_{22}+U_{23}+U_{24}, \end{array}$$

where *k*^∗^ is a factor depending on the form of the cross section (for regular section which is equal to 1.2), *e* is the distance from the centroidal axis to the neutral axis, *e* = *R* − *b*/[ln(*R* + *b*/2) − ln(*R* − *b*/2)], and *θ* is the angle from axis of *x* to point *P*. Therefore, we can separately calculate the energy of *U*_2*i*_(*i* = 1, 2, 3, 4) as

(11) $$\begin{array}{c} U_{21}=\int \frac{M_{P}^{2}}{2AEe}d\theta =\frac{1}{2AEe}\int {\left[-F_{11}\left(y_{2}-y_{1}\right)+F_{12}\left(x_{2}-x_{1}\right)+M_{1}\right]}^{2}d\theta ,\\ U_{22}=\int \frac{{kV}^{2}R}{2AG}d\theta =\frac{{kF}^{2}R}{2AG}\int \cos^{2}\left(\theta +\alpha_{1}\right)d\theta ,\\ U_{23}=\int \frac{N^{2}R}{2AE}d\theta =\frac{F^{2}R}{2AE}\int \sin^{2}\left(\theta +\alpha_{1}\right)d\theta ,\\ U_{24}=-\int \frac{M_{P}N}{AE}d\theta =\frac{F}{AE}\int \left[-F_{11}\left(y_{2}-y_{1}\right)+F_{12}\left(x_{2}-x_{1}\right)+M_{1}\right]\sin\left(\theta +\alpha_{1}\right)d\theta , \end{array}$$

where the coordinates of point *B*(*x*_1_, *y*_1_) and point *P*(*x*_2_, *y*_2_) are

(12) $$\begin{array}{c} x_{1}=-R\cos\alpha_{3},\\ y_{1}=R\sin\alpha_{3},\\ x_{2}=-R\cos\theta ,\\ y_{2}=R\sin\theta . \end{array}$$

For part III, we can calculate the normal force *F*_31_ and tangential force *F*_32_ as

(13) $$\begin{array}{c} F_{31}=F\sin\alpha_{4},\\ F_{32}=F\cos\alpha_{4}, \end{array}$$

where *α*_4_ is the angle between *F* and horizontal in this part, which can be calculated as *α*_4_ = *α*_1_ + *θ*_0_ − *pi*. Therefore, the stresses in this part are

(14) $$\begin{array}{c} \sigma_{3x}=\frac{F_{31}}{A}-\frac{F_{32}xy}{I}+\frac{M_{2}y}{I},\\ \sigma_{3y}=0,\\ \tau_{3xy}=\frac{3}{2}\frac{F_{32}}{A}\left[1-{\left(\frac{2y}{b}\right)}^{2}\right], \end{array}$$

where *M*_2_ is the moment in point *C*, which is equal to *F*_12_*R*(cos*α*_3_ − cos*θ*_0_) − *F*_11_*R*(sin*θ*_0_ − sin*α*_3_) + *M*_1_. *θ*_0_ is the angle of the rib. The energy of part III can be calculated as

(15) $$\begin{array}{c} U_{3}=\int \frac{1}{2}\sigma_{3}\varepsilon_{3}dV+\int \frac{\tau_{3xy}^{2}}{2G}dV=\int {\left(\frac{F_{31}}{A}-\frac{F_{32}xy}{I}+\frac{M_{2}y}{I}\right)}^{2}\frac{1}{2E}dV+\int \frac{9}{4}\frac{F_{32}^{2}}{{GA}^{2}}{\left[1-{\left(\frac{2y}{b}\right)}^{2}\right]}^{2}dV=\frac{F_{31}^{2}L_{2}}{2EA}-\frac{F_{32}^{2}L_{2}^{3}}{6EI}-\frac{F_{32}M_{2}L_{2}^{2}}{2EI}+\frac{M_{2}^{2}L_{2}}{2EI}+\frac{3F_{32}^{2}L_{2}}{5GA}. \end{array}$$

Therefore, we can find the Δ*θ* as

(16) $$\begin{array}{c} \Delta \theta =\frac{2\left(U_{1}+U_{2}+U_{3}\right)}{T}. \end{array}$$

And the stiffness can be obtained as

(17) $$\begin{array}{c} k=\frac{T}{\Delta \theta }. \end{array}$$

According to these formulations, we could make the conclusion that the stiffness of *k* is in proportion to the ribs number of *N*, modulus elasticity of *E*, and thickness of *h*.

### 3.2. FEM Static

**Figure 4:**
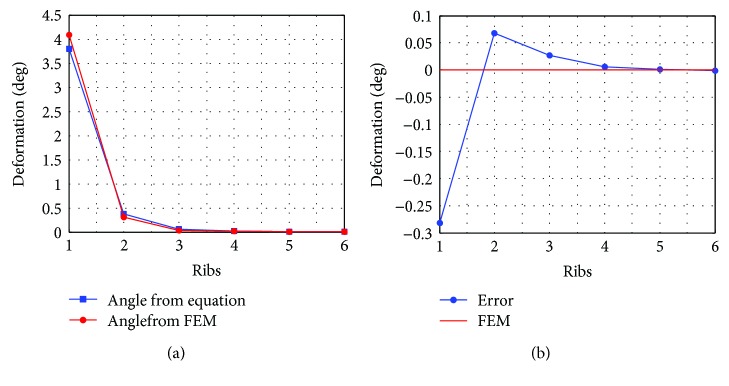
Relationship between deformation and the number of ribs: (a) deformation; (b) error.

The six typical configurations are analyzed by using ANSYS. In the analysis, the mesh type is hexahedron element, and each model comprises approximately 35,000 elements and 140,000 nodes. A fixed constraint is applied to the inner flange, and a torque load of 1 Nm is applied to the outer flange. The analysis results are listed in Table 3. The relationship of displacement with those 6 configurations as FEM and formulae is shown in Figure 4.

The displacement of the applied torque of the elastic element was reduced from 2.1381 mm to 0.0025852 mm when the number of ribs increased. The maximum stress of 157.23 MPa was less than the material yield stress of 500 MPa. Therefore, in this condition, the stress was not the core issue to consider. Finally, the 2-rib structure was found to be optimal because the deformation of one rib was beyond the measurement range of RPI-131, while the deformation of others was significantly small. Furthermore, the displacement of 0.1627 mm was detectable using the RPI-131.

### 3.3. FEM Optimization

Optimization was measured to determine the optimal structure. The topology optimization to achieve the lightest weight was conducted using ANSYS with the reliability constraints and stress limitations. An efficient method for topology optimization is reducing the quality and volume to make the model approach the optimized target. In this case, the mechanical performance should be partly retained.

**Figure 5:**
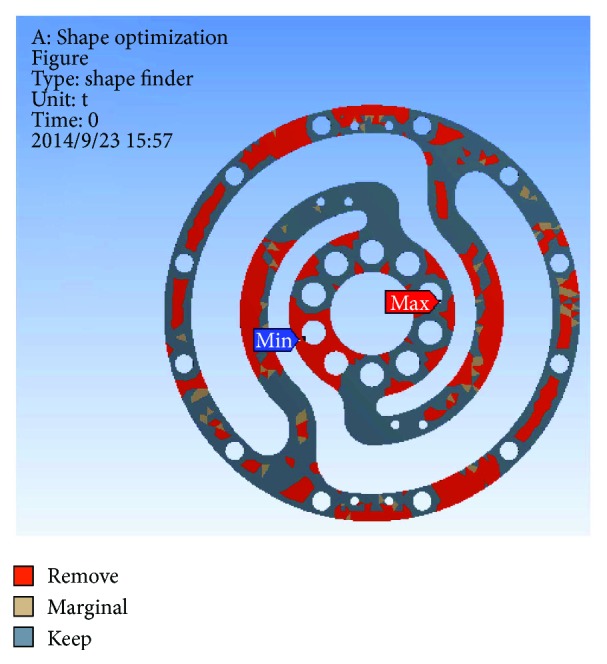
Result of optimization.

In the analysis, the solid element was accepted. Approximately 90,000 elements and 160,000 nodes were produced in the meshing. Similarly, a fixed constraint was applied to the inner flange, and the torque was loaded at the outer flange. The quality of the target optimization was set to half.  Figure 5 shows that the red area could be removed without significantly affecting the performance in mechanics.

**Figure 6:**
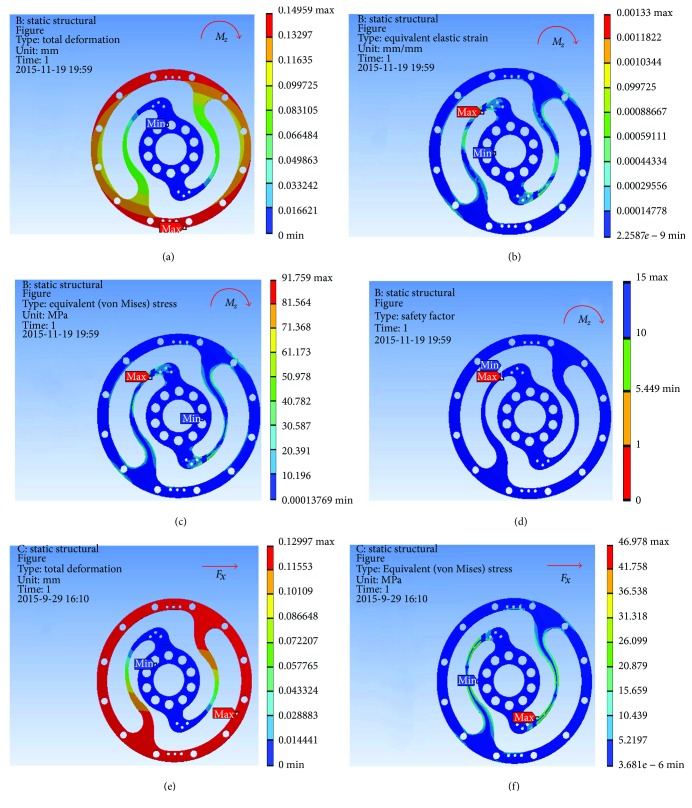
FEA results of optimized structure: (a) deformation from *M*_*z*_; (b) strain from *M*_*z*_; (c) stress from *M*_*z*_; (d) factor of safety from *M*_*z*_; (e) deformation from *F*_*x*_; (f) stress from *F*_*x*_.

Based on the result of optimization, we finally acquired the ideal configuration by modelling with the method of eccentric circle to approach the optimization results. To evaluate the performance of the optimized 2-rib configuration, the static analysis was reconducted. In the analysis, the solid element was also accepted, and approximately 54,000 elements and 98,000 nodes were produced. The same torque load and fixed constraint were applied on the outer and inner flanges, respectively. As this sensor is immune to the loads of *M*_*x*_, *M*_*y*_, *F*_*y*_, and *F*_*z*_ (the deformation from these loads could not be detected by the photointerrupters), the influence by *F*_*x*_ needs to be considered. In the analysis, the force load (15 N) and fixed constraint were also applied on the outer and inner flanges, respectively.  Figure 6 shows the analysis results of the optimized 2-rib structure. The comparison of performances between the original and optimized structures is listed in Table 4.

According to (a) to (f) in Figure 6, we can obviously find that under the load of *M*_*z*_, the deformations detected by the two photointerrupters are identical. While, under the load of *F*_*x*_, the deformations detected by the two photointerrupters are opposite. Therefore, the error caused by *F*_*x*_, in a certain range, could be eliminated through the simple signal process such as be average. Eventually, the designed sensor could detect the torque of *M*_*z*_ without influence from other loads.

**Figure 7:**
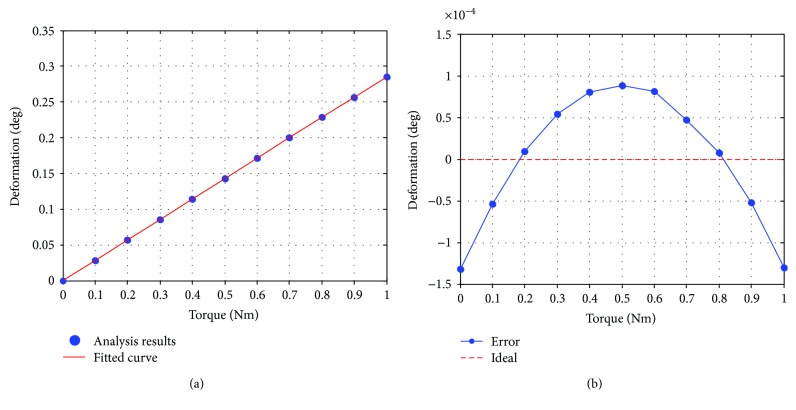
Simulation in the range of 0~1 Nm: (a) relationship of torque and displacement; (b) error.

And through the topology optimization, the results show that the displacement was reduced by 7.01%, and the mass was reduced by 11.15%; meanwhile, the factor of safety was also reduced by 29.63%. Theoretically, the linearity of this sensor can be seen in Figure 7.

## 4. Experiments and Results

The performances of linearity, hysteresis, and repeatability are important in evaluating the feasibility of the design of torque sensor.

### 4.1. Experiment Preparation

**Figure 8:**
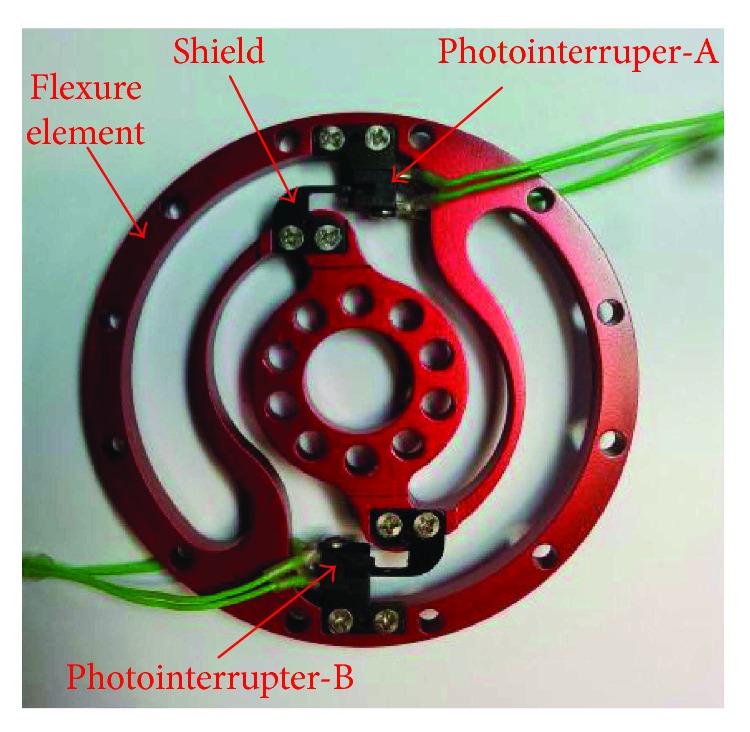
Manufactured torque sensor.

**Figure 9:**
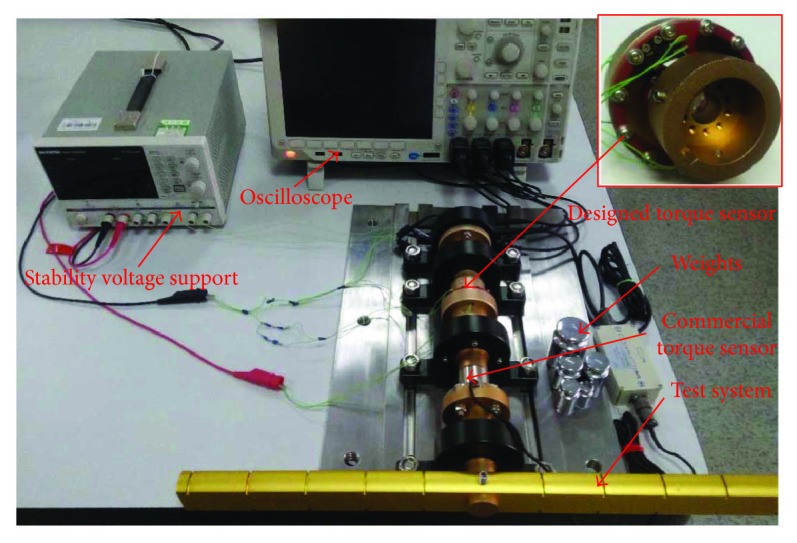
Calibrate test system.

**Figure 10:**
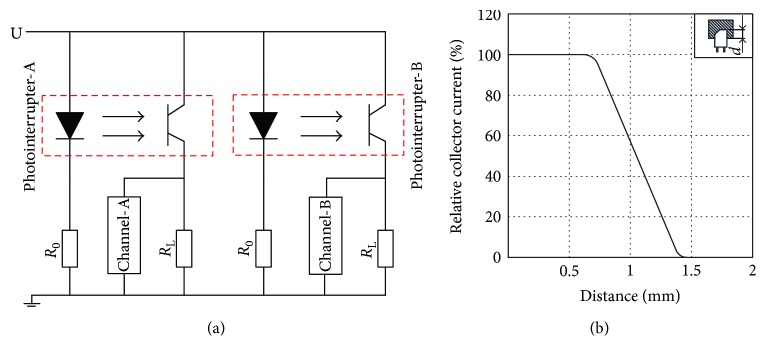
Experiment circuit: (a) working principle of photointerrupter; (b) measure range.

The sensor, discussed in this paper, is manufactured with a computer numerical control milling machine (Figure 8), and the test system (Figure 9) is built to study its performances.  Figure 10 shows the established experiment circuit. The photointerrupter is composed of an infrared light emitting diode (LED) at one side and a transistor (detector) at the other side (shown in Figure 10(a)). The graph in Figure 10(b) shows there is a linear relationship between the distance and the current of the collector. According to this linear relationship, acquiring the applied torque size by detecting shield displacement would be successful. Considering the sensitivity, *U* = 10 **V** and *R*_*L*_ = 10 **k***Ω* were selected [13]. *R*_0_ = 833 *Ω* was chosen for *I* ≈ (*U* − *U*_*d*_)/*R*_0_ = (10**V** − 1.16 **V**)/833*Ω* = 10.4**mA** (where *U*_*d*_ ≈ 1.16 **V** that is the voltage drop caused by LED). Channels A and B were linked to the oscilloscope channels 1 and 2, respectively. In this kind of circuit, the maximum output voltage was 9.8 V, and the output voltage in the initial position was approximately 4.5 V.

### 4.2. Linearity

**Figure 11:**
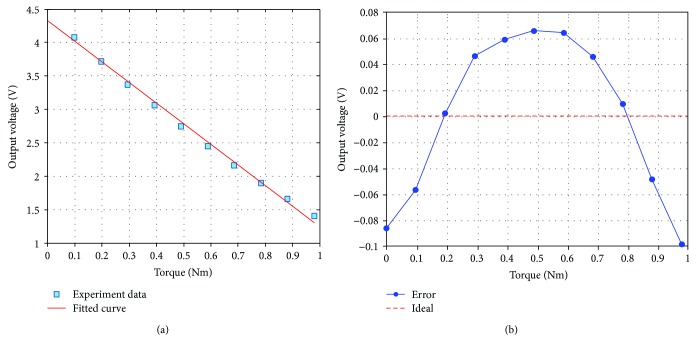
Experiment results of applied torque and resulting output: (a) linearity; (b) error.

Linearity describes the degree offset between the actual line and ideal straight line [31, 32]. The torque is achieved (from 0 Nm to 0.98 Nm) by placing load at the lever of the test system, and the increased torque is 0.098 Nm. The experiment is conducted more than 50 times to ensure the acquired data truly reflect the performance of the sensor. The experiment data are recorded in Table 5, and the graphs of linearity and error are drawn in Figure 11. With using polyfit function of MATLAB, the relationship between displacement and applied torque can be built as

(18) $$\begin{array}{c} \Delta =-3.0198T+4.3250, \end{array}$$

where Δ is the output voltage and *T* is the applied torque size. The nonlinearity and torque sensitivity are expressed as

(19) $$\begin{array}{c} \gamma_{L}=\frac{\Delta L_{\max}}{FS}\times 100\%=\frac{0.0986}{4.325-1.3052}\times 100\%=3.27\%,\\ S=\frac{\Delta Y}{\Delta X}=\frac{4.4118-1.4038}{0.98}=3.07\;V/Nm, \end{array}$$

where Δ*L*_max_ is the maximum error, FS is the full scale, Δ*X* is the full range of torque input change, and Δ*Y* is the corresponding change in the output voltage.

### 4.3. Hysteresis

**Figure 12:**
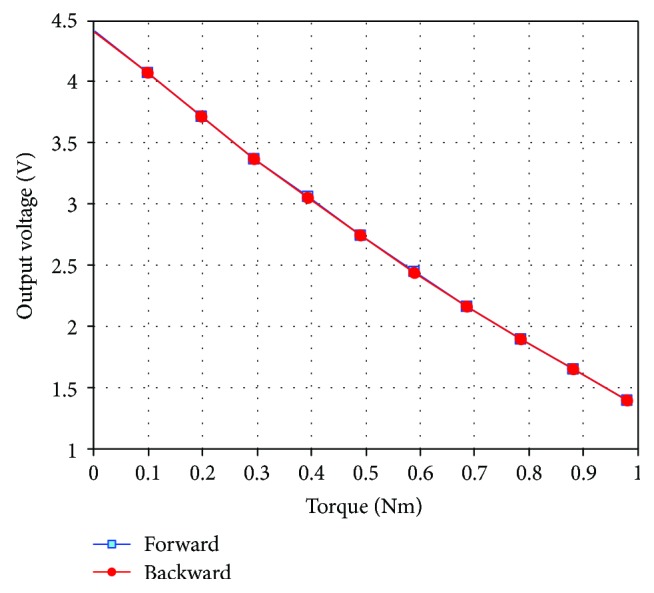
Hysteresis curve.

Hysteresis describes the degree of misalignment between the input and output with the forward and backward loads. The hysteresis experiment was tested by applying torque in ascending and descending manners at 0.098 Nm. The results of the hysteresis experiment are shown in Table 6 and Figure 12. The graph of the hysteresis curve was drawn using MATLAB. With a maximum offset Δ*H*_max_ of 0.006 V, the hysteresis is calculated as

(20) $$\begin{array}{c} \gamma_{H}=\frac{\Delta H_{\max}}{FS}\times 100\%=0.2\%. \end{array}$$

### 4.4. Repeatability

**Figure 13:**
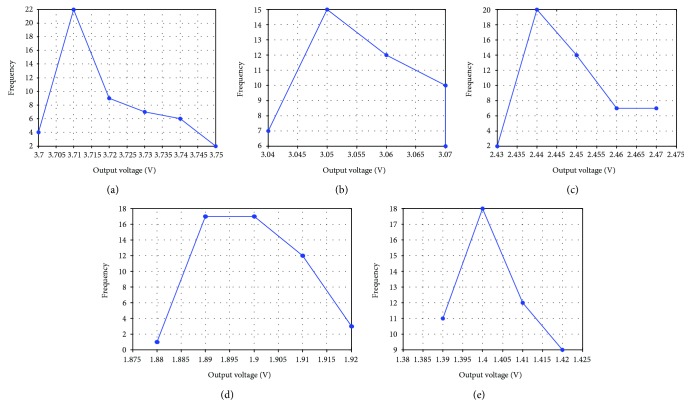
Repeatability curve: (a) torque 0.196 Nm; (b) torque 0.392 Nm; (c) torque 0.588 Nm; (d) torque 0.784 Nm; (e) torque 0. 98 Nm.

Repeatability is usually necessary when an unsteady system is used to measure errors. Each graph in Figure 13 shows the results of the applied torque repeatedly applied for 50 times. The graph means that with the application of the same torque, the frequency of equal output voltage can be detected. For the maximum span Δ*R*_max_ of 0.07 V, the repeatability can be calculated as (21). The graph presents the detected frequency of equal output voltage applying the same torque.

(21) $$\begin{array}{c} \gamma_{R}=\frac{\Delta R_{\max}}{FS}\times 100\%=2.32\%. \end{array}$$

### 4.5. Factor of Safety

Generally, that the most effective method to evaluate the property of factor of safety is to conduct the experiment of overload. The experiment is conducted with 0.5 Nm increment of load from 1 Nm to 5 Nm. In each load, the experiment is conducted about 3 times with maintaining load at least 5 minutes. After removing the load, the output voltage of photointerrupter is recorded in Table 7. From the results, we can clearly find that in the range of 1 Nm to 4.5 Nm, this sensor could operate normally, while at the torque size of 5 Nm, it has been failed. Therefore, the factor of safety of this sensor should be about 4.5.

### 4.6. Evaluation

The performance of the designed torque sensor is shown in Table 8. Recently, the research of flexible spring applied to the torque sensor is less. Obviously, the linearity is a little insufficient which may be caused by its principle of sensing element or the especially flexure spring. However, compared with other optical torque sensors [12–14], the torque sensor designed in this paper has a wide measure range and a small diameter, while the thickness of only 4 mm is the best of them. At the same time, it is equally satisfied with a light mass and a high safety factor; hence, it has a compact and smart structure that can be perfectly applied to biomimetic robot joints.

## 5. Conclusion

In this paper, a highly reliable optical torque sensor is developed for biomimetic robot joints in extreme embedded applications. To ensure the high reliability, the structure is designed based on a flexure spring, which is most commonly used in many fields. The thickness is reduced to 4 mm, the weight is 16 g, and the factor of safety is 4.5. The torque sensor is very small for being used in a biomimetic joint with a maximum output torque size of 1 Nm obviously. The linearity, sensitivity, repeatability, and hysteresis are 4.31%, 2.06 V/Nm, 2.67%, and 0.2%, respectively. In the future, using signal process to promote its performance and studying multiforce/moment condition with similar size are worth considering. Improving the measurement dimension of the torque sensor and its application capacity in the new generation of biomimetic robot joints are our next vision and priorities.

## Figures and Tables

**Table 1:** Parameters of the torque sensor.

Item	Parameter	Item	Parameter
Diameter	60 mm	Mount	11 mm
Thickness	4 mm	Inner/outer flange	*ϕ* 3 mm/*ϕ* 2.5 mm
Hole	12 mm		

**Table 2:** Six typical configurations.

Number of ribs	1	2	3
Configuration	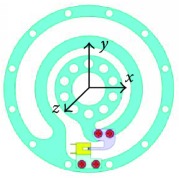	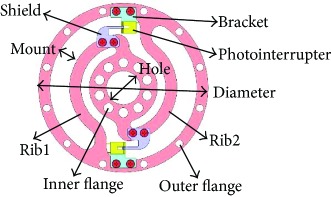	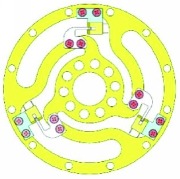
Multidetection	*M*_*z*_/*F*_*x*_	*M*_*z*_, *F*_*x*_	*M*_*z*_, *F*_*x*_, and *F*_*y*_
Number of ribs	4	5	6
Configuration	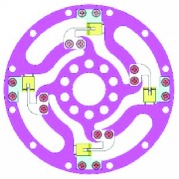	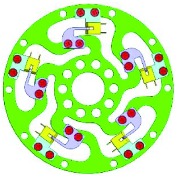	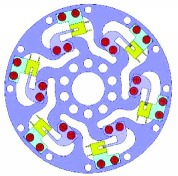
Multidetection	*M*_*z*_, *F*_*x*_, and *F*_*y*_	*M*_*z*_, *F*_*x*_, and *F*_*y*_	*M*_*z*_, *F*_*x*_, and *F*_*y*_

**Table 3:** Results of finite element analysis.

Ribs	Structure	Displacement	Stress	Results
1	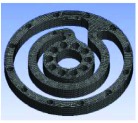	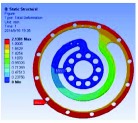	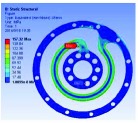	Max displacement 4.0835 deg Max stress 157.32 Mpa
2	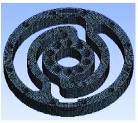	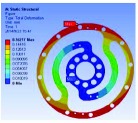	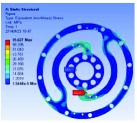	Max displacement 0.3097 deg Max stress 65.627 Mpa
3	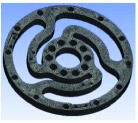	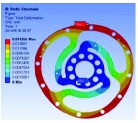	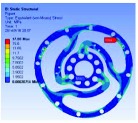	Max displacement 0.0272 deg Max stress 17.55 Mpa
4	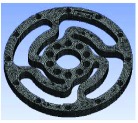	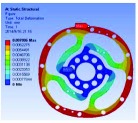	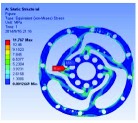	Max displacement 0.0134 deg Max stress 11.767 Mpa
5	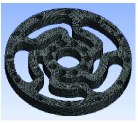	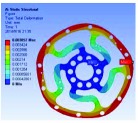	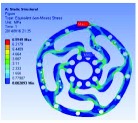	Max displacement 0.0074 deg Max stress 6.9949 Mpa
6	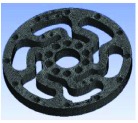	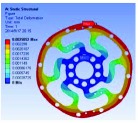	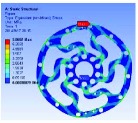	Max displacement 0.0049 deg Max-stress 5.8602 Mpa

**Table 4:** Comparison of performances of original and optimized structure.

Item	Original	Optimized
Mesh	34,000 elements, 136,000 nodes	60,000 elements, 11,000 nodes
Displacement	0.16086 mm	0.14959 mm
Max stress	64.567 MPa	91.759 MPa
Max strain	0.00089679 mm	0.00133 mm
Factor of safety	7.7439	5.449
Mass	15.442 g	13.72 g

**Table 5:** Experiment data.

Torque (Nm)	Minimum (V)	Maximum (V)	Average (V)	Calculate (V)	Error	% error
0	4.40	4.43	4.4118	4.3250	−0.0868	2.87
0.098	4.05	4.12	4.0800	4.0231	−0.0569	1.88
0.196	3.70	3.75	3.7190	3.7211	0.0021	0.70
0.294	3.35	3.40	3.3730	3.4191	0.0461	1.53
0.392	3.04	3.08	3.0586	3.1171	0.0585	1.94
0.49	2.73	2.77	2.7496	2.8151	0.0655	2.17
0.588	2.43	2.47	2.4494	2.5131	0.0637	2.11
0.686	2.15	2.19	2.1654	2.2112	0.0458	1.52
0.784	1.89	1.92	1.8998	1.9092	0.0094	0.31
0.882	1.64	1.67	1.6560	1.6072	−0.0488	1.62
0.98	1.39	1.42	1.4038	1.3052	−0.0986	3.27

**Table 6:** Results of hysteresis.

Torque (Nm)	Forward (V)	Backward (V)	Error (V)
0	4.410	4.407	0.003
0.098	4.079	4.079	0.000
0.196	3.717	3.717	0.000
0.294	3.372	3.370	0.002
0.392	3.060	3.054	0.006
0.49	2.746	2.746	0.000
0.588	2.446	2.441	0.005
0.686	2.160	2.160	0.000
0.784	1.894	1.892	0.002
0.882	1.650	1.649	0.001
0.98	1.398	1.398	0.000

**Table 7:** Data of overload experiment.

Torque (Nm)	1	1.5	2	2.5	3.0	3.5	4	4.5	5
1 (V)	4.41	4.40	4.40	4.41	4.42	4.41	4.40	4.42	4.41
2 (V)	4.41	4.42	4.39	4.40	4.42	4.40	4.41	4.42	4.35
3 (V)	4.40	4.40	4.39	4.41	4.42	4.40	4.41	4.41	4.21

**Table 8:** Performances of designed torque sensor.

Item	Optical torque sensor
Mass	16 g
Diameter	60 mm
Thickness	4 mm
Factor of safety	4.5
Linearity	3.27%
Sensitivity	3.06 V/Nm
Repeatability	2.32%
Hysteresis	0.2%
